# Efficacy of Human Tendon-Derived Extracellular Matrix in Achilles Tendon Regeneration

**DOI:** 10.3390/jcm15124716

**Published:** 2026-06-17

**Authors:** Seong Kyeong Jo, Kumaresan Sakthiabirami, Do Hyi Gu, Dae Hyung Lee, Yeji Choi, Dae Young Kim, Jae Hwang Song

**Affiliations:** 1 Department of Orthopedic Surgery, Konyang University Hospital, Daejeon 35365, Republic of Korea; joseongkyeong@gmail.com (S.K.J.); sakthikarthi.dentist@gmail.com (K.S.); 2 Department of Clinical Pathology, Konyang University, Daejeon 35365, Republic of Korea; gdh0218@naver.com; 3 Department of Pathology, Wonkwang University Hospital, Iksan 54538, Republic of Korea; 4 Advanced Medical Device R&D Center, HansBiomed Co., Ltd., Seoul 05836, Republic of Korea; leedh@hansbiomed.com (D.H.L.); yjchoi@hansbiomed.com (Y.C.); 5 Department of Pathology, College of Medicine, Konyang University, Daejeon 35365, Republic of Korea; ohmygod2631@hanmail.net

**Keywords:** Achilles tendon injury, human tendon-derived extracellular matrix (hT-ECM), extracellular matrix (ECM), tendon healing, collagen

## Abstract

**Background/Objectives**: Achilles tendon injuries exhibit slow and incomplete healing due to limited vascularity, cellularity, and a complex extracellular matrix (ECM). Biologically derived ECM-based therapies have emerged as promising strategies to enhance tendon repair by providing native biochemical cues. In this study, the efficacy of injectable human tendon-derived ECM (hT-ECM) was investigated using a rat Achilles tenotomy model. **Methods**: Percutaneous tenotomy of the right Achilles tendon in 50 rats was performed. The animals were subjected to either no treatment as a control group (CON) or hT-ECM treatment group (ECM). Tendon healing was evaluated at 1 and 6 weeks using biomechanical, histological, transmission electron microscopy, Western blot, and immunohistochemical analysis. **Results**: At 6 weeks, the ECM group exhibited higher load to failure (54.5 ± 15.0 N) than the CON group (28.6 ± 13.7 N) (*p* < 0.05). Histological evaluation revealed progressive restoration of the injured tendon in both groups over the healing period, with comparable results observed between the groups. Ultrastructural analysis revealed that the ECM group exhibited significantly increased collagen fibrils diameters (74.1 ± 19.1 nm) than the CON group (53.8 ± 13.8 nm) (*p* < 0.001). Western blot exhibited that collagen I (COL I) expression at 6 weeks was significantly higher in the ECM group compared with the CON group (*p* < 0.05). **Conclusions**: These outcomes suggest that injectable hT-ECM improves early repair responses in a rat Achilles tenotomy model. Molecular analyses suggested that the therapeutic effects of hT-ECM injection could be linked to enhanced collagen type I production. Based on the study, hT-ECM injection might be a good adjuvant option for the treatment of Achilles tendon injuries.

## 1. Introduction

Injuries to tendons and ligaments affect over 30 million people worldwide each year, creating major socio-economic and clinical challenges [1,2]. Among these soft tissue injuries, Achilles tendon rupture is the most common injury with a reported annual incidence of 18 per 100,000 people [3]. The Achilles tendon is the largest and most resilient tendon in the human musculoskeletal system, which connects the gastrocnemius and soleus muscles to calcaneal tuberosity [4]. It is a vital connective tissue that provides necessary support for movement and joint stability. However, there are limitations in its healing capacity due to its low vascularity and cellularity, which result in poor regenerative potential [5]. Achilles tendon injuries range from mild strain and tendinopathy to complete ruptures and are frequently associated with sports participation, occupational overuse, and age-related degeneration. They often cause delayed recovery and result in functional impairment [6]. The current nonoperative treatments such as immobilization, physiotherapy, and corticosteroid injections usually provide temporary symptom relief, and they often fail to restore tendon function [7]. Although frequent recurrence and retear underscore the importance of operative treatment, large surgical incisions may increase the risk of infections, adhesions, and wound complications, limiting their overall benefit [8]. Hence, conservative treatment combined with effective biologically driven adjuvant therapies to promote tendon healing is needed.

Recently, several biological and mechanically supportive approaches have been developed to enhance the regenerative capacity of the tendon [9]. They include strategies such as mesenchymal stem cells (MSCs), platelet-rich plasma (PRP), polyribonucleotide (PDRN), autografts, acellular dermal matrices (ADMs), and atelocollagen [10,11,12]. Among them, extracellular matrix (ECM)-based biomaterials have gained attention in regenerative medicine, as they preserve native structural proteins and bioactive cues essential for cell adhesion, differentiation, and tissue regeneration.

ECM-based materials are derived from allogenic or xenogeneic origin [13,14]. In general, ECM-based materials are typically derived from the skin tissue or soft tissue of xenografts [15,16,17]. However, xenogeneic materials carry potential risks of immune rejection and disease transmission, while allogenic types minimize these concerns and are thus more suitable for clinical applications. Each tissue possesses a distinct ECM composition and exhibits tissue specificity, which provides an optimal biochemical microenvironment via cytokines and factors that regulate cell behavior and tissue repair [18,19]. Consequently, supplementing injured tissues with their native ECM components is expected to promote faster and more regeneration. Human tendon-derived ECM (hT-ECM) offers a distinct advantage by closely mimicking the compositional, biochemical, and structural characteristics of native human tendon tissue, potentially enabling superior host integration and functional regeneration. Nevertheless, the application of hT-ECM as an injectable therapy for Achilles tendon healing has received relatively little attention and warrants further in vivo investigation. Therefore, the present study aims to evaluate the regenerative potential of hT-ECM injection in a rat model of Achilles tendon rupture, assessing its effects regarding structural healing, molecular remodeling, collagen fibrillogenesis, and biomechanical restoration.

## 2. Materials and Methods

### 2.1. Injection Material

**Figure 1:**
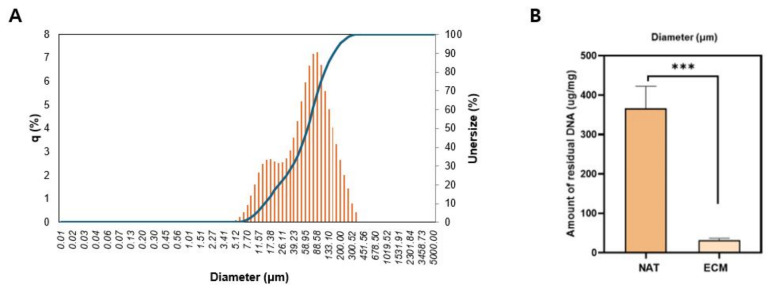
Characteristics of human tendon-derived ECM (hT-ECM). (**A**) Particle size distribution of hT-ECM, (**B**) decellularization efficiency (residual DNA quantity) (*** *p* < 0.001).

A micronized hT-ECM (Re:tendon™ HansBiomed, Seoul, Republic of Korea) product, injection type, was used in this study. This product has been approved by the Korean Ministry of Food and Drug Safety. The manufacturing process of Re:tendon™ was carried out as follows. Human tendon tissues (Achilles, tibialis posterior, and patellar tendon) were thawed and trimmed to remove any residual fat and soft tissues, then subjected to freeze-drying. The dried tendon tissues were subsequently delipidated and decellularized by immersing them in dimethyl ether, followed by overnight degassing at room temperature to remove any residual solvent. The amount of residual solvent was evaluated according to US EPS 8060C:2006 (GC/MS) to verify the effectiveness of the degassing process. The processed tissues were cryogenically milled, and the resulting ECM powder was sieved using a standard test sieve (sieve opening size = 100 µm,425 µm) in accordance with ASTM and ISO 330 standards to ensure uniform particle distribution and effective decellularization. The particle size distribution of the obtained ECM powder was analyzed by dispersing the powder in 0.9% saline, followed by sonification and measurement was carried out using the laser diffraction method. The results confirmed a particle size ≤ 400 µm, with the mean particle size 62.8 µm (Figure 1A). Residual DNA in the ECM powder was quantified using a DNA purification kit (Qiagen, Hilden, Germany) according to the manufacturer’s protocol. Thus, the absorbance of DNA samples (*n* = 5) was measured at 260 and 280 nm with a Bio Tek microplate reader (Winooski, VT, USA) to determine the A260/280 ratio. The calculated mean residual DNA content of the native tendon and hT-ECM was 336.2 µg/mg and 32.1 µg/mg, respectively (*p* < 0.001, Figure 1B). In addition, the amino acid composition was determined by a high-speed amino acid analyzer (Hitachi, Hitachinaka, Japan). A 100 mg ECM powder sample was hydrolyzed in 10 mL of 6 N HCl at 110 °C for 22 h. After hydrolysis, the sample was dried under reduced pressure to remove HCL. The hydrolysate was filtered for further purification using a 0.20 µm pore size syringe filter. The filtered hydrolysates were diluted in a 1:10 ratio with filtered deionized water and placed into sample vials. Table 1 displays the amino acids and their contents.

The tendon powder was rehydrated in 0.9% saline, thoroughly mixed, and dispensed into sterile syringes. The loaded syringes were blister-packed and sterilized using electron beam irradiation. Re:tendon™ is manufactured at ECM concentrations ranging from 3% to 20% depending on the intended clinical applications, with a 10% formulation used in this study. A 10% ECM concentration was selected for this study to reflect the composition of ECM-based products commonly used in clinical practice, thereby enhancing the translational relevance of the experimental design.

### 2.2. Animal Model and Surgical Procedure

The Institutional Animal Care and Use Committee (IACUC) approved the animal experimental protocols in accordance with the Guide for the Care and Use of Laboratory Animals [20]. A total of fifty 10-week-old male Sprague–Dawley rats (Samtako Bio, Osan, Republic of Korea) were obtained from a licensed laboratory animal supplier. Animals were sheltered under standard laboratory conditions with free access to food and water. Animals were divided into two groups: a non-injection group (control group, CON, *n* = 25) and a Re:tendon™ injection group (ECM injection group, ECM, *n* = 25). The operative procedure was carried out based on previous studies [10,21,22]. In brief, surgical procedures were performed under inhalation anesthesia using isoflurane with a mixture of oxygen and nitrogen [10]. Each rat was placed in the prone position on a sterile operating platform. A percutaneous tenotomy was performed at the right Achilles tendon. A 2 mm stab incision was made on the lateral aspect using a NO. 11 scalpel blade, followed by fascial incision to expose the Achilles and Plantaris tendon. A complete transverse tenotomy was then performed perpendicular to the orientation of the collagen fibers, approximately 5 mm proximal to the calcaneal insertion site [10]. In the control group, no material was administered, whereas in the ECM group, 0.03 mL of Re:tendon™ (10% formulation) was injected directly into the tenotomy site using a syringe [10]. Native tendon tissue was harvested from the right Achilles tendon of a separate uninjured animal. The skin incision was then closed using 3-0 nylon sutures (Ethicon, San Angelo, TX, USA). After surgery, all rats were housed and allowed free ambulation without immobilization. At a designated time point (1 and 6 weeks), animals were sacrificed under anesthesia (isoflurane). Following confirmation of loss of reflexes and complete unconsciousness, euthanasia was performed by an overdose of urethane administered via intraperitoneal injection (1.5–2.0 g/kg). Death was confirmed by the absence of respiration and cardiac activity prior to tissue harvesting.

**Figure 2:**
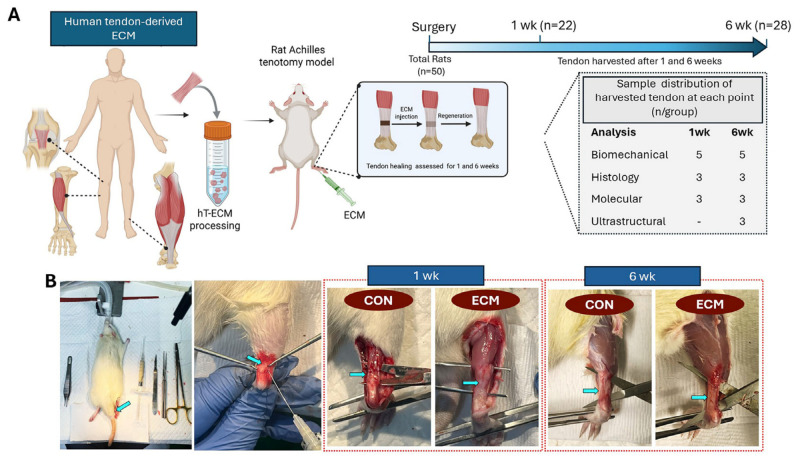
Study outline. (**A**) Schematic image illustrating experimental workflow of the present study. (**B**) Rat Achilles tenotomy model and ECM injection. Gross images of CON and ECM groups at 1 and 6 weeks (blue arrow indicates the surgical, injection and healing site).

A total of 50 rats were included in the study and evenly assigned to the two time points (1 and 6 weeks post-surgery). At each time point, harvested tendons were assigned to specific analyses as follows: biomechanical (*n* = 5), histological (*n* = 3), ultrastructural morphological assessment using transmission electron microscopy (TEM, Hitachi, Tokyo, Japan, *n* = 3), and molecular analyses (*n* = 3) [10]. In addition, the right Achilles tendon was used as the experimental site, while the contralateral left Achilles tendon served as the native (NAT) control. Figure 2A,B display schematic images illustrating the experimental workflow of the present study and surgical procedure.

### 2.3. Biomechanical Analysis

At 6 weeks post-surgery, biomechanical evaluation of the harvested tendon was performed using a tensile testing machine (500-N EZ-SX Texture Analyzer; Shimadzu, Kyoto, Japan). The tendon was excised along with the attached calcaneus and musculotendinous segment. The width and the thickness of each sample were measured with a digital vernier caliper (SD500-150PRO; Sincon, Shanghai, China) to calculate the cross-sectional area (CSA, mm^2^). Under the assumption of an elliptical geometry, the CSA at the central region of the callus was calculated from two perpendicular diameter measurements, with a representing the major axis and b the minor axis (CSA = π × a × b/4). The tendon was secured proximally at the musculotendinous junction using serrated metal grips, while the calcaneus was clamped distally to ensure stable fixation. Uniaxial tensile loading was applied at a constant rate of 0.1 mm/s until failure, and the corresponding load displacement curve was recorded. The ultimate load to failure (N), stiffness (N/mm), and stress (N/mm^2^) were obtained from the data, with stiffness determined from the linear region of the load displacement curve prior to failure. Stress was calculated as load to failure divided by CSA. All data were processed using Trapezium software (v2.05, Shimadzu, Kyoto, Japan).

### 2.4. Histology Analysis

Achilles tendon specimens were harvested at 1 and 6 weeks post-surgery. The collected samples were processed using routine histological procedures. Tissues were fixed in 4% paraformaldehyde for 24 h, followed by dehydration through graded ethanol series. Subsequently, the samples were immersed in xylene for clearing and embedded in paraffin. Paraffin blocks were sectioned at 5 µm thickness using a rotary microtome (RM2255; Leica, Wetzlar, Germany). For each group, three tissue sections were randomly selected, mounted on the glass slides, deparaffinized in xylene, and rehydrated through a descending alcohol gradient [10]. The tissues were stained with hematoxylin and eosin (H&E), Masson’s trichome (MT), and Alcian blue (AB). Histology evaluation was performed using the modified Bonar scoring system [21,23], assessing five parameters including cell morphology, vascularity, cellularity, collagen fiber arrangement, and ground substance. Each parameter was graded from 0 (normal tendon) to 3 (severely abnormal), and the total score was obtained as the sum of all five parameters. The total Bonar score for each specimen was obtained by adding the five characteristic grades, along with an additional 2.5 points assigned for the presence of intratendinous calcification and adipocytes. Histologic evaluation was conducted by a single experienced observer and was not independently blinded.

### 2.5. Transmission Electron Microscopy (TEM)

TEM is used to investigate ultrastructural changes during tendon healing. Achilles tendon tissue harvested at 6 weeks (approximately 1 × 1 mm) was fixed in 2.5% glutaraldehyde at 4 °C, followed by washing with phosphate buffer (0.1 M) and post-fixation in 1% osmium tetroxide. The samples were dehydrated with the series of ethanol, embedded in Embed-812 epoxy resin, and polymerized at 60 °C for 48 h. Ultrathin sections were cut with a diamond knife on an RMC ultramicrotome (RMC Boeckeler, Tucson, AZ, USA) and subsequently stained with 2% uranyl acetate and 1% lead citrate. The ultrastructure was visualized using a HT7700 TEM (Hitachi, Tokyo, Japan) at 80 kV. Collagen fibril diameter and fibrillar organization were analyzed in three samples per group, measured using ImageJ software 1.54 (NIH, Bethesda, MD, USA).

### 2.6. Western Blot Analysis

Western blot analysis was performed on Achilles tendon tissues harvested at 1 and 6 weeks post-surgery. Proteins were extracted from the samples using PRO-PREP™ lysis buffer (iNtRON Biotechnology, Seongnam, Republic of Korea) and centrifuged at 15,000 rpm for 20 min at 4 °C [10]. Protein concentrations were measured using the bicinchoninic acid (BCA) assay (Thermo Fisher Scientific, Waltham, MA, USA). Equal protein quantities were subjected to SDS-PAGE and subsequently transferred onto the polyvinylidene fluoride (PVDF) membranes. Following transfer, the membrane was blocked with 5% skim milk in tris-buffered saline containing Tween 20 (TBST) and then incubated overnight at 4 °C with the following primary antibodies: collagen type I (COL I), collagen type III (COL III), vascular endothelial growth factor (VEGF), transforming growth factor-β1 (TGF-β1), transforming growth factor-β3 (TGF-β3), and β-actin (1:2000; Invitrogen and Santa Cruz Biotechnology, Dallas, TX, USA). Subsequently, membranes were washed and treated with secondary antibodies (1:10,000; Invitrogen) for 2 h at room temperature. Protein bands were observed using chemiluminescence reagents (Thermo Fisher scientific) and quantified with ImageJ software 1.54 (NIH, Bethesda, MD, USA). Relative protein expression levels were normalized to β-actin.

### 2.7. Immunohistochemistry (IHC)

Immunohistochemical staining was performed to assess the expression of COL I, COL III, and VEGF in regenerated Achilles tendon tissues. In addition, CC chemokine receptor type 7 (CCR7) and cluster of differentiation 163 (CD163) were assessed to evaluate the immune cell distribution and activation. Paraffin-embedded sections were deparaffinized in xylene, rehydrated through a graded ethanol series, and subjected to antigen recovery using Target Retrieval solution by incubating the slides overnight at 40 °C. Endogenous peroxide activity was blocked by treating the sections with 3% hydrogen peroxide for 30 min at room temperature. Sections were then incubated overnight at 4 °C with the respective primary antibodies, followed by secondary antibodies (goat anti-rabbit IgG and goat anti-mouse IgG) with the incubation for 2 h at room temperature. Subsequently, the slides were treated with an avidin–biotin–peroxidase complex for 1 h 30 min and developed with DAB and H_2_O_2_ for 2 min. Finally, the sections were counterstained with H&E and examined under a microscope for qualitative analysis.

### 2.8. Statistical Analysis

Sample size was determined via power analysis in G*Power (v3.1.9.7) based on prior studies using similar methods [21]. Based on data from our earlier work, a total of six rats (three per group) were estimated to be sufficient to detect a 3.2 ± 0.9 difference in histological scores, assuming a power of 80% and an alpha level of 0.05. Means ± standard deviations were reported with corresponding *p* values. Comparisons between the two groups were tested using Student *t* test with *p* < 0.05 considered significant [10]. Statistical analyses were performed in SPSS (v22.0; IBM, New York, NY, USA).

## 3. Results

### 3.1. Biomechanical Testing

**Figure 3:**
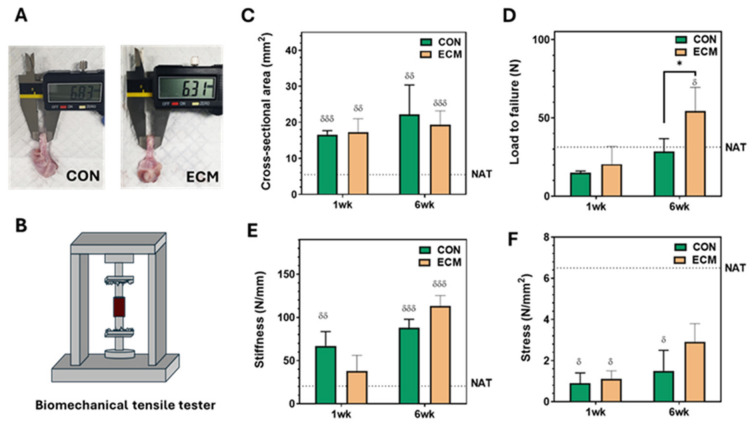
Biomechanical evaluation of tendon healing. (**A**) Images showing measuring of tendon width and thickness using digital vernier caliper in CON and ECM group prior to mechanical testing. (**B**) Schematic illustration of tensile testing using a uniaxial mechanical tester. (**C**–**F**) Biomechanical outcomes at 1 and 6 weeks post-surgery including (**C**) cross-sectional area, (**D**) load to failure, (**E**) stiffness, and (**F**) stress. The dotted line indicates result for the native tendon (NAT). Error bars indicate standard deviations. Statistically significant difference between the COL and ECM tendons (* *p* < 0.05) at the same time point and between the COL or ECM tendons and NATs at the same time point (δ *p* < 0.05, δδ *p*< 0.01, δδδ *p* < 0.001).

Gross examination at 1 and 6 weeks (Figure 1B) revealed tendon continuity in all specimens. Biomechanical testing showed a progressive increase in tendon strength from 1 to 6 weeks (Figure 3C–F). Cross-sectional area (Figure 3C) and stiffness (Figure 3E) increased from 1 to 6 weeks in the CON and ECM groups, and both groups remained higher than the native tendon (NAT). Load-to-failure values showed an increase over time, with the ECM group (54.5 ± 15.0 N) exhibiting a significantly higher load to failure than the CON group (28.6 ± 13.7 N) (*p* < 0.05) at 6 weeks (Figure 3D). Similar trends were displayed in the stress values. Although neither group achieved outcomes equivalent to those of the native tendon, the ECM group demonstrated earlier restoration of stress compared with the CON group, showing results comparable to the native tendon at 6 weeks.

### 3.2. Histological Analysis

**Figure 4:**
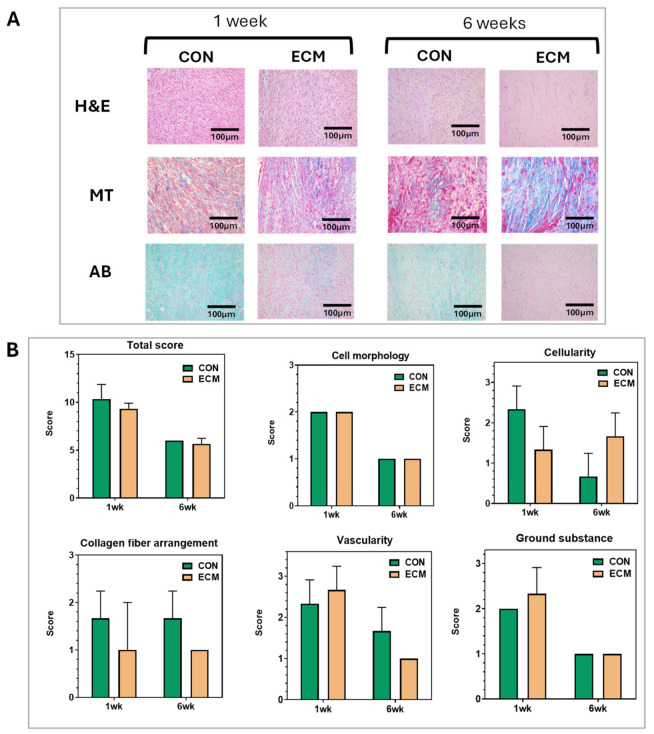
Histological evaluation of the present study. (**A**) Microscopic image of CON and ECM group with H&E, Masson’s trichrome (MT), and Alcian blue (AB) staining after 1 and 6 weeks post-surgery, respectively (200× magnification). (**B**) Quantitative scoring of histological outcomes using modified Bonar score including total score, cell morphology, cellularity, collagen fiber arrangements, vascularity and ground substance.

Histological evaluation revealed progressive restoration of the injured tendon, with improved tissue organization and maturation in both groups over the healing period (Figure 4A,B). Both groups showed comparable results in the modified Bonar score. At 1 week, both groups exhibited disrupted collagen architecture, increased cellularity and ground substance, indicating the early stage of healing. At 6 weeks, both groups demonstrated improved collagen bundle organization, with tenocytes adopting an elongated spindle-shaped morphology and a noticeable reduction in ground substance content, exhibiting maturation of tendon healing. No evidence of infection was observed in either group.

### 3.3. Transmission Electron Microscopy (TEM) Analysis

**Figure 5:**
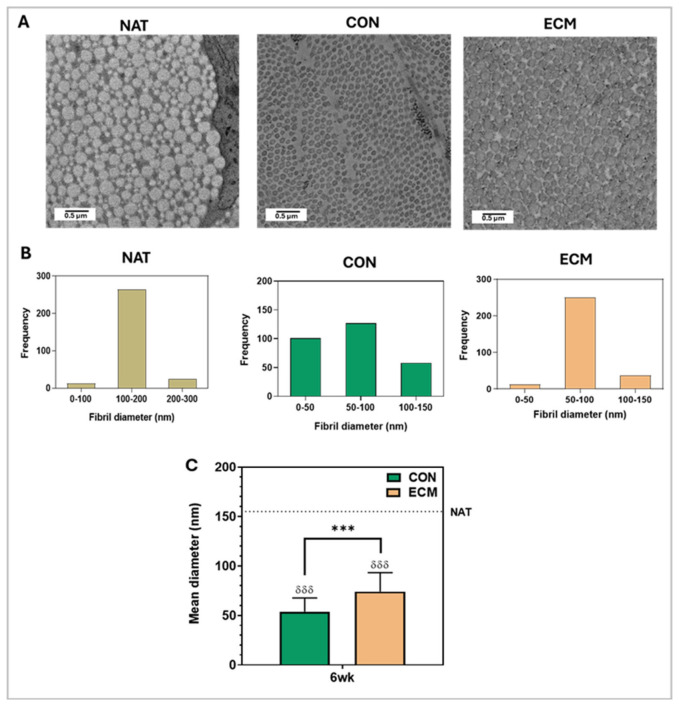
Transmission electron microscopy (TEM) analysis at 6 weeks. (**A**) TEM images of native (NAT), CON, and ECM group, respectively. (**B**) Frequency of collagen fibril diameters and (**C**) mean diameter of collagen fibrils for NAT, CON, and ECM group, respectively. Statistically significant difference between the CON and ECM (*** *p* < 0.001), and between the CON or ECM tendons and NATs (δδδ *p* < 0.001).

Cross-sectional TEM images of collagen fibrils were obtained from both groups after 6 weeks (Figure 5A,B). The distribution of collagen fibril diameters within each group was not uniform. The ECM group exhibited significantly higher collagen fibril diameters (74.1 ± 19.1 nm) than the CON group (53.8 ± 13.8 nm) (*p* < 0.001). However, both groups displayed smaller diameters than those of the native tendon (154.9 ± 0.8 nm) (*p* < 0.001).

### 3.4. Western Blot

**Figure 6:**
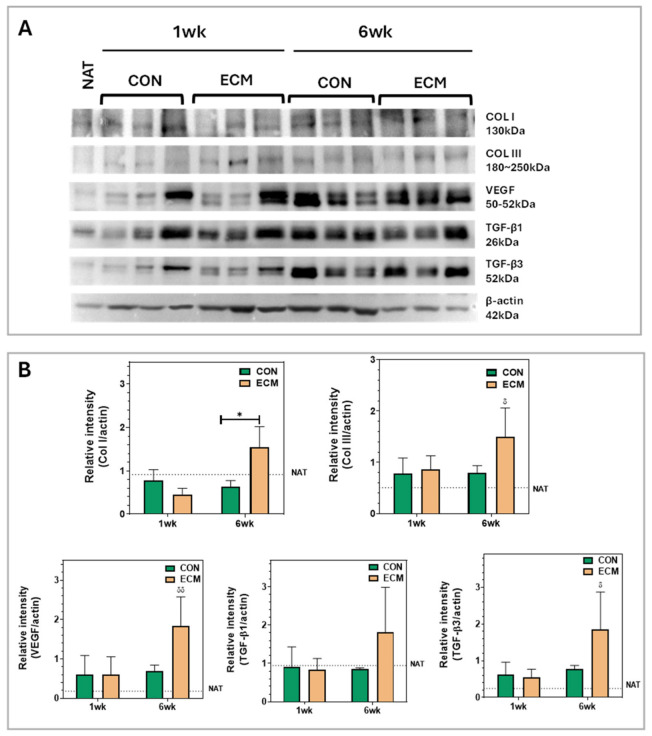
Western blot analysis of the present study. (**A**) Band images show the expression of collagen type I (COL I), collagen type III (COL III), vascular endothelial growth factor (VEGF), transforming growth factor-β1 (TGF-β1), and TGF-β3. (**B**) Quantitative analysis of each protein expression level, which was normalized with β-actin. For each protein, the ratio of band intensity to β-actin intensity was calculated. Values represent the means (y-axis) for each group’s samples (x-axis). Error bars indicate standard deviations. Statistically significant difference between the COL and ECM tendons (* *p* < 0.05) and between the COL or ECM tendons and NATs at the same time point (δ *p* < 0.05, δδ *p* < 0.01).

Western blot analysis at weeks 1 and 6 demonstrated time-dependent modulations of tendon-associated proteins including COL I, COL III, VEGF, TGF-β1, and TGF-β3 (Figure 6A,B). Notably, COL I expression at 6 weeks was significantly higher in the ECM group compared with the CON group (*p* < 0.05). Although other proteins such as COL III, VEGF, TGF-β1, and TGF-β3 displayed a tendency to increase over time in both groups, no significant difference was observed between the CON and ECM groups for these markers. Compared with the native tendon, proteins such as COL III, VEGF, and TGF-β3 showed significantly increased expression in the ECM group at 6 weeks.

### 3.5. Immunohistochemistry (IHC)

**Figure 7:**
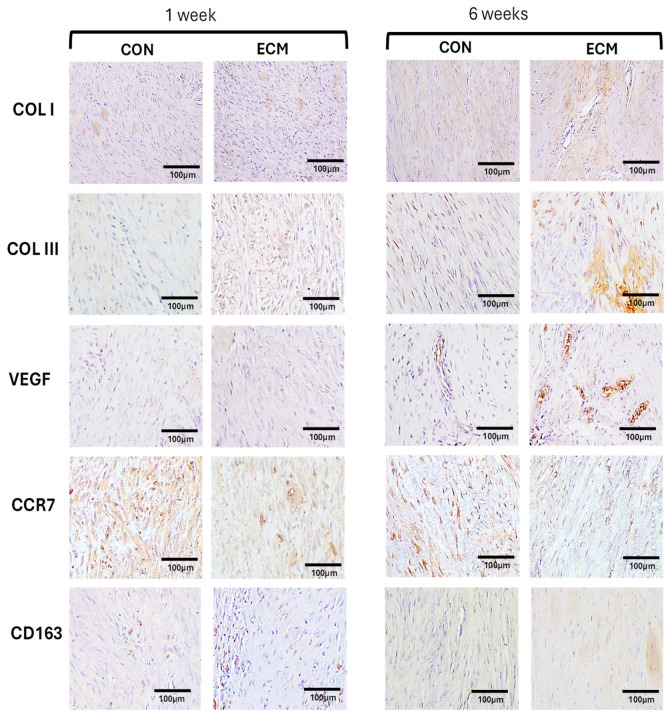
Immunohistochemical staining of COL I, COL III, VEGF, CCR7, and CD163 at 1 and 6 weeks in CON and ECM group, respectively (200× magnification).

At 6 weeks, COL I expression in the ECM group was higher than that in the CON group (Figure 7). Meanwhile, the staining results showed that higher expression of both COL III and VEGF was found in the ECM group at 6 weeks post-surgery. Furthermore, immune cell markers CCR7 (M1-type, pro-inflammatory macrophages) displayed a decreasing trend from 1 to 6 weeks in both groups, whereas CD163 (M2-type, anti-inflammatory macrophages) displayed a gradual increase over the time period.

## 4. Discussion

Recent advances in tendon tissue engineering highlight the importance of biomaterials that replicate the native ECM with its biochemical components [2,7,19]. Human tendon-derived ECM is uniquely suited for this purpose, as it holds enriched native bioactive cues such as COL I, proteoglycans, glycosaminoglycans, growth factors, and signaling molecules, which promote matrix remodeling. Despite its promising potential, the use of hT-ECM for Achilles tendon regeneration remains insufficiently explored. To address this gap, the present study evaluated the regenerative potential of an injectable hT-ECM in a rat model.

The most important finding of the present study is that the protein level of COL I was markedly elevated in the ECM group compared with that in the CON group at 6 weeks, as confirmed by Western blot (Figure 6) and IHC (Figure 7). In addition, the ECM group demonstrated enhanced Achilles tendon regeneration, with greater biomechanical strength and increased collagen fibril diameter compared with the CON group. Overall, these results support our hypothesis that hT-ECM promotes healing of the Achilles tendon.

Biomechanical testing (Figure 3) revealed progressive improvement in tensile properties from 1 to 6 weeks in both groups, consistent with the expected transition from the inflammatory phase to the remodeling phase of tendon repair [24]. The significantly higher load-to-failure value observed in the ECM group at 6 weeks indicates superior structural restoration than the CON group. The injected ECM scaffold might have contributed to collagen fibrillogenesis, resulting in improved biomechanical outcomes. Histological analysis (Figure 4) showed progressive tissue remodeling and matrix organization in both groups over time, consistent with the natural sequence of tendon healing. The transition of enlarged tenocytes toward an elongated spindle-shaped morphology and the reduction in ground substance reflect the gradual replacement of the early reparative matrix with more organized collagen bundles, indicating the onset of remodeling and maturation [25]. The improved collagen alignment and reduced cellularity over time suggest that fibroblast-to-tenocyte differentiation and matrix deposition were advancing toward a matured tendon-like phenotype.

In TEM analysis, the native tendon exhibited thick and densely packed collagen fibrils typical of mature tendon architecture, whereas the regenerated groups showed smaller and more heterogeneous fibril diameters, reflecting incomplete maturation (Figure 5). The non-uniform distribution of fibril diameters in both CON and ECM indicates ongoing collagen fibrillogenesis, which indicates the early-to-intermediate healing phase [2,26]. The significantly larger fibrils in the ECM group compared with the CON group suggest that the ECM-based treatment promoted more effective fibril assembly and matrix organization, possibly through enhanced tenocyte activity and ECM deposition [27]. The larger fibrils observed in the ECM group strongly support the more favorable mechanical and histological findings, suggesting enhanced fibrillogenesis and improved collagen cross-linking during the healing process. These results align with earlier studies demonstrating that collagen supplementation or ECM-mimicking scaffolds promote fibril thickening and hierarchical matrix assembly [10,26]. Despite this improvement, both groups remained structurally inferior to the native tendon, consistent with limited cross-linking and incomplete hierarchical reorganization even during the 6-week period [28]. Continued maturation, mechanical loading, and ECM remodeling over longer durations are likely required to restore the native fibrillar alignment and mechanical integrity characteristic of healthy tendon tissue [22,28].

Western blotting was performed to evaluate the expression of tendon-related and inflammatory proteins. Protein expression profiles demonstrated dynamic ECM remodeling throughout the healing period. At 6 weeks, COL I expression was significantly higher in the ECM group than the CON group (Figure 6), suggesting that the injected ECM scaffold effectively enhanced collagen synthesis and matrix maturation [29]. In contrast, COL III expression showed no significant differences between the groups, suggesting a comparable early-phase repair process dominated by immature collagen deposition. VEGF expression exhibited an upward trend in the ECM group at 6 weeks, implying increased angiogenic activity near the repair site. Enhanced VEGF expression has been reported to promote neovascularization and nutrient diffusion during tendon regeneration [30,31]. TGF-β3 expression gradually increased over the 6 weeks, with the ECM group showing significantly higher levels than the native tendon at 6 weeks, suggesting a shift toward more organized and scar-less healing [32]. Collectively, these findings suggest that the injected ECM scaffold may contribute to a pro-regenerative microenvironment that supports collagen fiber formation [33].

IHC assessments validated and supported the aforementioned Western blot findings. COL I expression was stronger in the ECM-treated group at 6 weeks, consistent with enhanced collagen synthesis and ECM organization (Figure 7). Across the experimental period, both groups demonstrated a temporal reduction in CCR7 levels alongside a progressive elevation of CD163 levels from 1 to 6 weeks. Comparative analysis revealed no statistically meaningful differences between the two groups in either CCR7 or CD163 expression at the evaluated time points. Consistent with earlier reports in tendon injury research, pro-inflammatory M1 macrophages are primarily observed during the initial phase, followed by later predominant M2 macrophages, a pattern that was likewise confirmed in this investigation [34,35]. The absence of significant differences in macrophage polarization between groups suggests that hT-ECM primarily influences structural matrix regeneration rather than altering upstream inflammatory pathways.

This study provides comprehensive in vivo demonstrations that injectable hT-ECM enhances early Achilles tendon repair. Another notable advantage of the present work lies in the comprehensive evaluation strategy, which incorporated biomechanical testing, histologic assessment, ultrastructural analysis, and molecular investigations to characterize the healing response following ht-ECM administration. When considered as an adjunct to conservative management, delivering ht-ECM in an injectable form appears particularly suitable, as this approach avoids the need for surgical skin incisions, unlike patch-, sponge-, or scaffold-type applications.

Several limitations should be acknowledged in the present study. To begin with, the follow-up period was limited to 1 and 6 weeks, and further studies with extended observation periods are necessary to clarify the early (days 1–3) and long-term (>6 weeks) effects of hT-ECM on tendon regeneration. The histologic evaluation was not conducted in a blinded fashion. In addition, unlike standard clinical practice in human patients, postoperative immobilization was not applied in this animal model. Finally, the exact biological pathways through which hT-ECM injections modulate molecular signaling, cellular activity, and ECM remodeling during tendon repair have yet to be fully elucidated. Future studies incorporating gene expression analyses may help to better define these underlying mechanisms.

## 5. Conclusions

The present study demonstrated that hT-ECM treatment significantly improved the key parameters of tendon healing. hT-ECM-treated tendons exhibited a distinct progression toward remodeling, marked by superior biomechanical properties, larger fibril diameters, and increased COL I expression. These findings highlight that hT-ECM-based scaffolds can provide regenerative potential in tendon tissue repair. Based on the current evidence, injectable ECM-based materials may represent a promising therapeutic strategy for enhancing regeneration after acute tendon injuries, including Achilles tendon rupture. Nevertheless, further studies with longer follow-up periods are needed to confirm these effects.

## Figures and Tables

**Table 1:** Amino acids and their contents.

No	Amino Acids	Content (%)
1	Asp	6.0
2	Thr	1.9
3	Ser	3.6
4	Glu	9.8
5	Gly	23.0
6	Ala	10.0
7	Cys	0.4
8	Val	2.0
9	Met	0.9
10	Ile	0.8
11	Leu	3.3
12	Tyr	0.5
13	Phe	2.2
14	Lys	3.1
15	NH3	0.7
16	His	0.8
17	Arg	7.4
18	Hypro	9.4
19	Pro	14.4
Total		100

## Data Availability

The data presented in this study are available in the article.
